# Feasibility of three-dimensional (3D) balanced steady-state-free-precession (bSSFP) myocardial perfusion MRI at 3 Tesla using local RF Shimming with dual-source RF transmission

**DOI:** 10.1186/1532-429X-15-S1-P23

**Published:** 2013-01-30

**Authors:** Roy Jogiya, Andreas Schuster, Arshad Zaman, Yasmine Samaroo, Eike Nagel, Sebastian Kozerke, Sven Plein

**Affiliations:** 1 Kings College London, London, UK; 2 Leeds University, Leeds, UK; 3 ETH Biomedical Engineering, Zurich, Switzerland

## Background

Three-dimensional myocardial perfusion MRI offers better myocardial coverage than conventionally used two-dimensional methods. bSSFP three-dimensional myocardial perfusion MRI at 3 Tesla potentially offers further improvement of signal characteristics and may enhance the use of three-dimensional myocardial perfusion MRI for clinical application.

## Methods

Twenty-five healthy volunteers and 2 patients were included upon written informed consent and local ethics committee approval. Dynamic contrast-enhanced 3D bSSFP perfusion imaging was performed on a 3 Tesla MRI scanner equipped with dual-source RF transmission technology (MultiTransmit; Philips Healthcare, The Netherlands).

## Results

**Figure 1:**
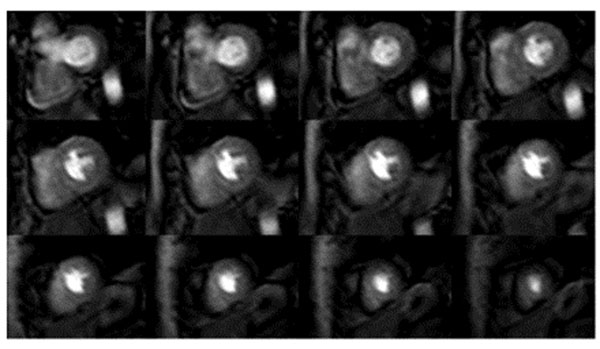
Volunteer example of 3D balanced steady state free precession (bSSFP) acquisition

Local RF Shimming with dual source RF transmission significantly improved B1 field homogeneity (P=0.0107). For bSSFP perfusion imaging, it allowed a reduction of TR from 3.4 to 2.2 ms at the same flip angle. Image quality was similar for TFE and bSSFP but there were more artefacts for bSSFP (Figure 1).

**Figure 2:**
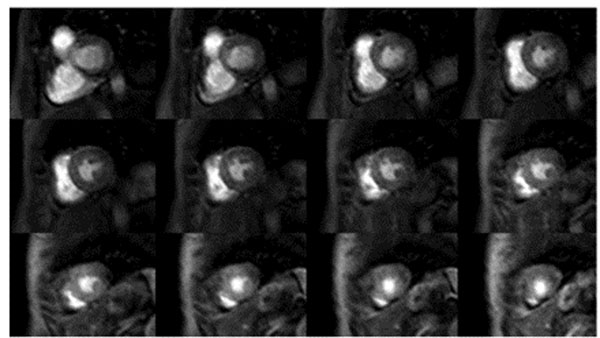
Volunteer example of 3D spoiled gradient echo (TFE) acquisition

Compared with an equivalent 3D spoiled gradient echo method (TFE), mean SNR was (30.4 vs 24.4, respectively, P=0.24), but signal homogeneity measured in the myocardium was improved (14.98% vs 11.15%, respectively, p=0.015).

## Conclusions

Three-dimensional bSSFP myocardial perfusion MRI using local RF Shimming with dual-source RF transmission at 3 Tesla is feasible with improved signal characteristics. Image artifacts however remain an important limitation.

## Funding

Prof. Plein is funded by British Heart Foundation fellowship FS/10/62/28409 and receives research grant support from Philips Healthcare.

Prof. Kozerke receives funding from the Swiss National Science Foundation (grant number CR3213_132671/1) and research support form Bayer (Switzerland) AG. Prof. Nagel receives grant support from Bayer Healthcare and Philips Healthcare.

